# Classification of Soybean Pubescence from Multispectral Aerial Imagery

**DOI:** 10.34133/2021/9806201

**Published:** 2021-08-04

**Authors:** Robert W. Bruce, Istvan Rajcan, John Sulik

**Affiliations:** Department of Plant Agriculture, University of Guelph, Guelph, ON, Canada

## Abstract

The accurate determination of soybean pubescence is essential for plant breeding programs and cultivar registration. Currently, soybean pubescence is classified visually, which is a labor-intensive and time-consuming activity. Additionally, the three classes of phenotypes (tawny, light tawny, and gray) may be difficult to visually distinguish, especially the light tawny class where misclassification with tawny frequently occurs. The objectives of this study were to solve both the throughput and accuracy issues in the plant breeding workflow, develop a set of indices for distinguishing pubescence classes, and test a machine learning (ML) classification approach. A principal component analysis (PCA) on hyperspectral soybean plot data identified clusters related to pubescence classes, while a Jeffries-Matusita distance analysis indicated that all bands were important for pubescence class separability. Aerial images from 2018, 2019, and 2020 were analyzed in this study. A 60-plot test (2019) of genotypes with known pubescence was used as reference data, while whole-field images from 2018, 2019, and 2020 were used to examine the broad applicability of the classification methodology. Two indices, a red/blue ratio and blue normalized difference vegetation index (blue NDVI), were effective at differentiating tawny and gray pubescence types in high-resolution imagery. A ML approach using a support vector machine (SVM) radial basis function (RBF) classifier was able to differentiate the gray and tawny types (83.1% accuracy and kappa = 0.740 on a pixel basis) on images where reference training data was present. The tested indices and ML model did not generalize across years to imagery that did not contain the reference training panel, indicating limitations of using aerial imagery for pubescence classification in some environmental conditions. High-throughput classification of gray and tawny pubescence types is possible using aerial imagery, but light tawny soybeans remain difficult to classify and may require training data from each field season.

## 1. Introduction

Plant breeding programs rely on phenotypic data generated from large-scale field trials to make informed decisions on germplasm advancement. This data is also used in cultivar registration and for determination of cultivar purity. Soybean pubescence (or trichome color) is a trait that is visually assessed, with an observer scoring plants or breeding plots for the trait class. This trait is expressed only at maturity when leaves have senesced, as the trichome colors are not visible when a full canopy cover is present. Soybean pubescence has three color classes: tawny, light tawny (near-gray), and gray. While gray and tawny types are visually distinguishable, the light tawny types are often misclassified due to the subtle color similarities between them and the other two classes.

The three classes of soybean pubescence are genetically distinct, with two genes, *T* and *Td*, interacting epistatically to control flavone synthesis [1, 2]. Tawny soybeans are *TT TdTd*, light tawny soybeans are *TT tdtd*, and gray soybeans are *tt TdTd* or *tt tdtd*. The *T* locus (Glyma.06g202300) encodes a flavonoid 3′-hydroxylase located on chromosome 6 [2]. The *Td* locus encodes a R2R3 MYB transcription factor (Glyma.03g258700) located on chromosome 3 which may interact with the two copies of flavone synthase II (FNS II-1 and FNS II-2) [1, 3].

Aerial imagery has been successfully deployed to assess a number of traits in soybean, with publications becoming more frequent in recent years with the decrease in price for imaging platforms and increasing availability of analytical methods to handle the data for plant researchers. Determination of soybean maturity has been effectively demonstrated from aerial imagery using several methods: partial least squares regression [4], random forest supervised machine learning [5], and convolutional neural networks [6]. Yield predictions from time-course imagery show promise for estimating final yield [5], and further work using deep neural nets continues this trend [7]. Aerial imagery has been used to assess iron deficiency chlorosis in soybeans [8] and to model canopy volume [9]. Soybean plot height and other biophysical characteristics have also been characterized from aerial imagery [10–12].

A range of indices is available for plant researchers to apply to their crops. Several simple band combinations can be used as simple ratio indices to characterize crop growth and development, while in recent years normalized difference indices have become more common in agricultural applications where they remain sensitive at lower levels of vegetation than ratio indices [13]. The blue normalized difference vegetation index (blue NDVI) has been used in remote sensing applications to characterize plant vegetation and water stress [14]. Custom indices based on exploratory data could be useful in dissecting traits not previously characterized with aerial imagery.

Machine learning (ML) is a popular approach to solving complex data problems, with numerous applications for agricultural research and data [15]. These applications can range from regression modeling for yield predictions to image analysis for field segmentation and more. Support vector machines (SVMs) are a class of reliable and effective ML methods widely used for classification [16]. Support vector machines function by defining a hyperplane at the boundaries between the classes of training data to generate the class separation boundaries for generalization [17]. The simplicity and power of SVMs make them convenient models for studying agricultural image data.

The objectives of this study were to understand the spectral characteristics of soybean pubescence from hyperspectral plot data and five-band aerial imagery, identify methodology to classify soybean pubescence from aerial imagery in an objective and high-throughput workflow, and assess model and index performance across growing seasons.

## 2. Materials and Methods

### 2.1. Field Trials

A field trial of 60 soybean plots (1.6 m × 5 m) with known pubescence (from multiple years of field validation) was planted at the Elora Research Station alongside the breeding plots at the University of Guelph (Guelph, ON, Canada). These plots consisted of 3 replications of 20 genotypes, which were studied as individual plots. These plots were imaged in 2019 and referred to as a Reference Test due to the previous, multiyear verification of pubescence type. A larger field test (200 genotypes with 2 replications, referred to as Full Test) was imaged over three growing seasons (2018, 2019, and 2020) at the Elora Research Station (43° 38′ 27.0456^″^ N, 80° 24′ 18.6948^″^ W), comprised of germplasm from a wide range of breeding activities. Fewer than 200 genotypes were present in each year, as genotypes with poor germination were not carried forward to the next year in the field tests (Table S1 for further plot details).

### 2.2. Hyperspectral Data Acquisition and Processing

The pubescence Reference Test plots (2019) were scanned with a UniSpec-DC Spectral Analysis System (PP Systems, Amesbury, MA, USA). Spectral values were captured between 310 nm and 1100 nm. The UniSpec-DC has a 3 nm spectral sampling distance, and the field of view (fov) was 23.5°. With the mature canopy, these measurements captured both plant and soil reflectance. Prior to plot measurement, the instrument was calibrated using a white reference panel (PP systems). Each plot was scanned twice, first from 1 m above the canopy and second 0.5 m above the canopy. The two plot scans were averaged prior to analysis. Spectral data under 344 nm was removed prior to analysis, due to no reflectance being recorded by the instrument below this threshold. A principal component analysis (PCA) was performed on the remaining hyperspectral data in three ways: plot-averaged data, simulated drone bandwidth from plot-averaged data (blue: 468 nm, 471.4 nm, 474.7 nm, 478 nm, and 481.4 nm; green: 554.7 nm, 558.1 nm, 561.4 nm, 564.7 nm, and 568.1 nm; red: 661.1 nm, 664.4 nm, 667.7 nm, 671.1 nm, and 674.4 nm; red edge: 710.8 nm, 714.1 nm, 717.4 nm, 720.7 nm, and 724.1 nm; and near-infrared: 836.3 nm, 839.6 nm, 842.9 nm, 846.2 nm, and 849.5 nm). All data were standardized before PCA.

### 2.3. Aerial Image Acquisition and Equipment

Soybean plots were imaged at R8 (harvest maturity) as they were ready for machine harvest [18]. The drones and sensors varied over the three seasons of imaging (Table 1). Flights ranged in height from 30 m to 100 m depending on weather conditions and flown according to manufacturer recommendations (gridded flight patterns with 75% side overlap distance, clear sky, and within 2 hours of solar noon and calibrated in Agisoft Metashape using a MicaSense calibration reflectance panel). For all image captures, a reference reflectance panel (MicaSense Inc) was captured with the sensor prior to flight for image calibration. The panel was spectrally flat with known reflectance.

In 2018 and 2019, the drone was a DJI M-210 with a MicaSense RedEdge3 multispectral camera (MicaSense Inc). For the 2019 pubescence image, a DJI M-210 was used with a MicaSense Altum, which primarily differs from the RedEdge3 in that each monochrome imager has 3 times as many megapixels. Both MicaSense cameras had the following band specifications in common: blue (475 nm center with 32 nm bandwidth), green (560 nm center with 27 nm bandwidth), red (668 nm center with 14 nm bandwidth), red edge (717 nm center with 12 nm bandwidth), and near-infrared (842 nm center with 57 nm bandwidth), with bandwidth described at full width half max [19] (Table 1). An additional image was analyzed in 2019 from a MicaSense Altum multispectral camera fitted with a Trimble APX-15-El Direct georeferencing system [20]. In 2020, DJI P4 Multispectral RTK drones (SZ DJI Technology Co) with included cameras were used for image capture with the following camera specifications: blue (450 nm ± 16 nm), green (560 nm ± 16 nm), red (650 nm ± 16 nm), red edge (730 nm ± 16 nm), and near-infrared (840 nm ± 26 nm) (Table 1). Several images had a thermal band (band 6); this band was removed prior to analysis. Images had varying ground sample distances (GSDs) depending on the sensors used for image capture and the altitude of the drone. The 2018 image had a pixel size of ~3.9 cm, the 2019 image had a pixel size of ~2.6 cm, and the 2020 image had a pixel size of ~3.6 cm (Table 1).

### 2.4. Orthomosaic Generation and Preprocessing

Image captures were processed into orthomosaics using Metashape (Agisoft LLC). The 2018 and 2019 images were georeferenced using ground control points (GCPs) in the form of white patio tiles placed throughout the field in the areas of interest for imaging. These GCPs had their positions captured using a Trimble AG-342 Global Navigation Satellite Sensor (GNSS) receiver real-time kinematic (RTK) corrections that provide cm-level accuracy. Image visualization and georeferencing were completed using QGIS 3.14 [21]. The 2020 images were georeferenced using the RTK functionality of the P4 Multispectral drone, so no CGPs were used in the georeferencing process. All images were projected and exported as UTM zone 17N. Plots were delineated manually in 2018 images, as the field was planted with a conventional planter. In 2019 and 2020, plots were delineated using plot positional data from an RTK-enabled plot planter and georeferenced aerial images [22].

Soil and nonsoybean vegetation pixels were masked from each image using an SVM (svmRadial) in the caret package [23] in R (R [24]). The raster package was used to load and manipulate image data within R [25]. The soil masks were applied to the whole-field images, resulting in only soybean pixels for downstream analysis.

### 2.5. Index Generation and Testing

The Jeffries-Matusita distance was calculated for all five image bands as well as a subset of the three visible image bands to determine whether the red edge and near-infrared bands improve separability between pairs of pubescence classes in the Reference Test 2019 image. The Jeffries-Matusita distance is a measure of spectral separability that is scaled to provide values between 0 and 2. A value of 0 indicates the least possible amount of separability between the spectral signatures of two classes whereas 2 suggests complete separability. Smaller separability values correspond with less accurate classification results [26].

To identify bands important to the color variation from the imaging data using a separate method, the loadings from the PCAs on the image data (with soil pixels removed) were analyzed from the 60 pubescence plots in the Reference Test Image 2019. From this loading data, it was determined that the red, blue, and near-infrared (NIR) bands had the best discrimination potential. Thus, several indices were tested including blue NDVI and ratio of red/blue:

(1) $$\begin{array}{c} \text{Blue NDVI}=\frac{\text{NIR}-\text{blue}}{\text{NIR}+\text{blue}},\\ \text{Red}/\text{blue}=\frac{\text{red}}{\text{blue}}. \end{array}$$

The average value for each of the listed indices was calculated for each plot from Reference Test Image in 2019, with soil removed prior to analysis. A separate analysis was attempted with soil pixels included, but effectiveness of the indices was reduced under these conditions and not further investigated. An ANOVA was conducted to determine whether each index was able to significantly differentiate the pubescence classes with a Tukey test to check for significant differences between each pair of classes (significant at *p* < 0.05). Confidence intervals (CIs) (95%) were calculated for the class mean of each index to provide guidance for generalizing these indices to other soybean plots. The midpoint between each CI was used to generate final index ranges for each class.

### 2.6. Machine Learning Pixel Classification

A machine learning (ML) approach was tested for classifying pubescence pixels from the soybean plots imaged. The ML was implemented in the caret package [23] in R (R [24]). A support vector machine (SVM) classifier with a radial basis function [27] was used for the modeling of soybean pubescence (the model is further referred to as SVMR). The radial basis function parameterizes a nonlinear transformation of the data, allowing a hyperplane to be generated for feature separation that would not be possible on untransformed data. Several datasets were tested as training data for the pubescence modeling, both larger scale including entire sections of each plot, and targeted, where individual pixels from a few plots from each pubescence type in the Reference Test 2019 image were used to train the model [28]. Training was completed using five separate 10-fold cross-validations. Training data pixels were randomly split from the testing data during the repeated cross-validations. The tuning parameter sigma was calculated to be 0.4376546, with a final *C* value of 512, resulting in an accuracy of 83.1% and kappa of 0.740. The trained model was used to classify the 60-plot pubescence Reference Image from 2019 and then further applied to the Full Field images from 2018, 2019, and 2020.

### 2.7. Testing the Indices and Machine Learning Models

To test the year-over-year generalization of the models (i.e., model inference), field images from 2018, 2019, and 2020 from various sensors and altitudes were analyzed. First, soil was masked as previously described; then, the various indices and SVM model were applied to the images without soil pixels. Plot extraction was conducted as previously described for the 2019 and 2020 images where precision planter GPS trip data was available. For 2018 images, plots were manually delineated. The QGIS zonal statistics tool was used to extract plot data using the plot outlines [22]. For ML outputs, both means and majorities were extracted, while for the indices, a mean per plot was the only statistic extracted from each plot. Plot statistics were joined to genotyping data to determine the accuracy of the methods for determining plot pubescence.

The Full Field images for the three seasons contained up to 366 plots (per season) for which single nucleotide polymorphism (SNP) genotype information was available (unpublished data from an ongoing PhD project of Cory Schilling). The SNPs corresponding to the T and Td loci were extracted from the dataset and used to determine the expected pubescence class of each plot. The T locus SNP used was on chromosome 6 at 18,732,972 and within the gene (Glyma.06g202300) while the Td locus SNP was on chromosome 3 at 45,299,226 (Glyma.03g258700) and approximately 1.6 kb upstream of the gene.

## 3. Results

**Figure 1:**
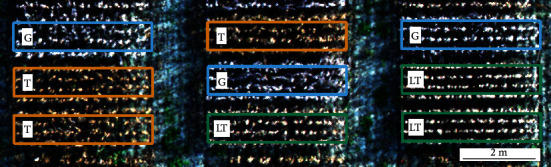
True color aerial image of soybean plots (Reference Test) at harvest maturity (R8) showing variation for pubescence color on October 15, 2019, at the Elora Research Station, University of Guelph, Guelph, ON, Canada. G = gray pubescence; LT = light tawny pubescence; T = tawny pubescence.

Soybean pubescence is typically observed from the ground level, where plant researchers and technicians visually score the trait as gray, tawny, or light tawny. Distinction between the tawny and light tawny classes is problematic from the ground level, where the subtle color differences can be masked by factors affecting human vision including nonuniform light, sun angle, and soil background. To further study these color differences, aerial images were collected from mature soybean research fields. From this aerial imagery, color differences were visible between the research plots under uniform lighting conditions (Figure 1).

A total of 60 soybean plots with known pubescence were grown at the Elora Research Station (University of Guelph) in 2019. These plots were seeded with genotypes which have been tested in multiple sites and years, in which the pubescence was recorded. These plots were used as the basis for assessing multiple methods of identifying variation in soybean pubescence color. Of the 60 plots, 24 had gray pubescence, 15 had light tawny pubescence, and 21 had tawny pubescence.

**Figure 2:**
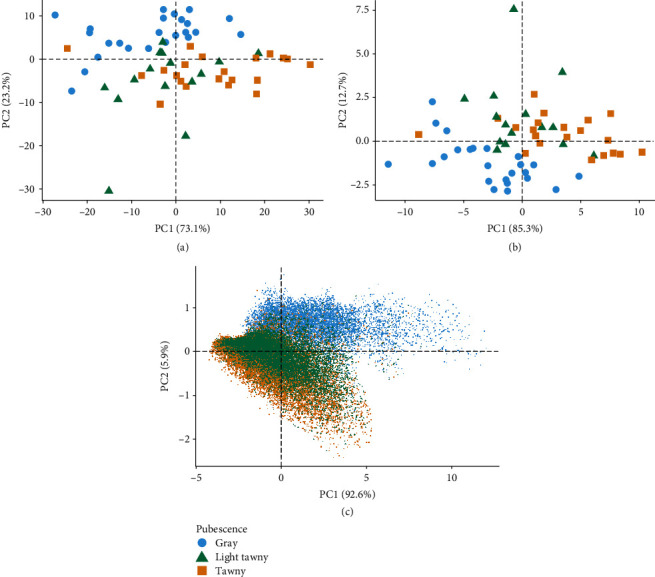
Principal component analysis (PCA) for (a) a plot average from 60 soybean plots with known pubescence from hyperspectral data (344 nm to 1100 nm at ~3 nm intervals) recorded with a UniSpec-DC reflectance spectrometer, (b) same data source as (a) except only wavelengths similar to a 5-band multispectral image were used, and (c) aerial imaging data from the same 60 plots from a 5-band multispectral camera, where each datapoint is a single pixel from within a plot of known pubescence. Plots were from the Reference Test 2019 Image at the Elora Research Station at the University of Guelph in 2019; all plots were at harvest maturity (R8).

A first step to studying the variation in pubescence was to gather plot-averaged hyperspectral data using a handheld UniSpec-DC reflectance spectrometer (PP Systems). From the hyperspectral data, a principal component analysis was conducted, resulting in PC1 accounting for 73.1% of the total variation and PC2 for 23.2% of the total variation. Linear separation of the three pubescence classes is identified using the hyperspectral data (Figure 2(a)). To understand the relationship of this data to a typical 5-band multispectral aerial image, bands were convolved from the hyperspectral data to mimic the band equivalent reflectance of the bandwidth and wavelengths present in 5-band aerial imagery. Using the simulated 5-band data, PC1 accounted for 85.3% and PC2 for 12.7% of the total variation, with groupings of the pubescence classes maintained in both datasets (Figure 2(b)). To confirm these trends in aerial image data, 36,039 pixels from the 60-plot pubescence test were extracted from 5-band aerial image data. A PCA of this pixel data resulted in 92.5% of the total variation on PC1 and 5.9% on PC2 (Figure 2(c)). Similar trends were seen as compared to the plot-averaged hyperspectral and simulated 5-band data; however, more overlap can be seen especially between the light tawny and tawny pixels from the image.

Pubescence class separability decreased for all class pairs when comparing five-band and three-band Jeffries-Matusita distances across the pubescence class pairs (Table 2). This result indicates diminished separability, especially between light tawny and gray. Therefore, all spectral bands were retained for the classification.

**Figure 3:**
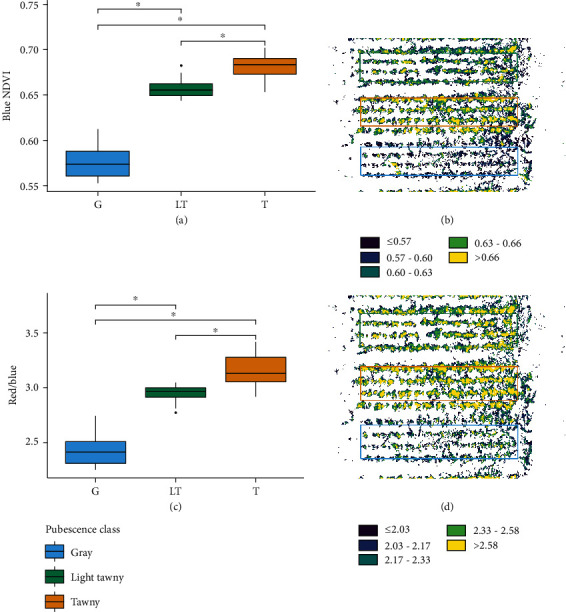
Average spectral index values for 60 soybean plots by pubescence type for (a) blue NDVI, (b) pseudocolor plot blue NDVI images, (c) red/blue index, and (d) pseudocolor plot red/blue images. All groups were significantly different from each other (ANOVA, *p* < 0.05, Tukey comparison).

Based on the PCA loading data from both 5-band imaging data (Table S2) and hyperspectral data (data not shown; similar trend to the 5-band data), the NIR and blue bands were found to be the most discriminatory in the datasets, so a blue NDVI was generated. To study the usefulness of an index approach without near-infrared data, an index of red over blue was also tested. For each index, soil pixels were removed from images of the Reference Test 2019 Image. Each index was calculated in QGIS for the plant pixels remaining with a final plot average kept for study. For all three indices, the class averages were significantly different from each other (ANOVA, *p* < 0.05) (Figure 3). In all three indices, the gray plots showed the greatest difference from the other two classes, while the light tawny and tawny plots were the least different. All three indices were able to separate the pubescence classes in the 60-plot pubescence dataset.

The blue NDVI and red/blue ratio had more overlap between the CIs for the light tawny and tawny classes, indicating a higher likelihood of misclassification of a plot. From the CIs, ranges were developed to classify plots based on the three tested indices (Table S3). In general, the indices all appear to be robust in the differentiation of gray pubescence from the other classes, while the light tawny and tawny classes may be more difficult to distinguish with overlapping CIs for all three indices.

A machine learning approach using an SVM classifier with a radial basis function was tested to classify pixels from the pubescence images. Instead of relying on statistical criteria for class membership, SVM classifiers exploit geometric criteria based on maximizing the margin between two classes [29]. The SVM classifier implemented in this study used the pairwise classification strategy for multiclass classification. The parameters were determined analytically where possible [23] and by grid search where analytical determination was not possible.

**Figure 4:**
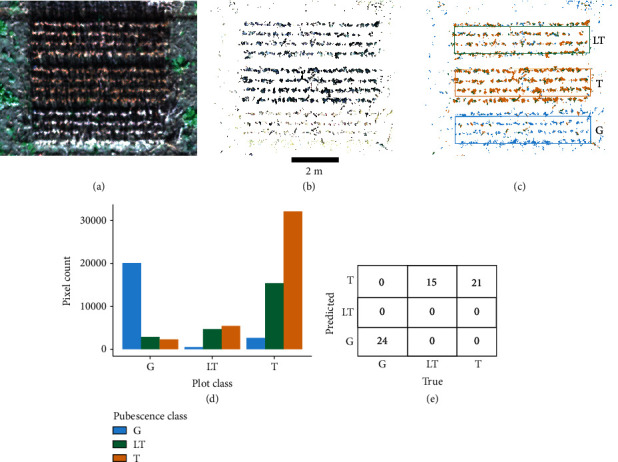
Machine learning classification results from the Reference Test 2019 Image 60-plot pubescence test using a support vector machine with a radial basis function in the caret package in R. (a) Three soybean plots from Elora Research Station in 2019. (b) Soil removal mask showing remaining plant pixels for classification. (c) Classified pixels from the ML algorithm with plot outlines showing the true pubescence of the plot. (d) Pixel counts for 60 pubescence plots from the ML output split by class. (e) Confusion matrix for the 60-plot pubescence test results by plot pixel majority.

Using the 60-plot Reference Test 2019 Image of the pubescence test, a range of training data was tested to identify a model with good fit (Figure 4). The final model chosen was an SVM with a radial basis function (for nonlinear separation of classes) with an overall accuracy of 83.1% and a kappa of 74.0%. The model was trained with 173 pubescence pixels from the 60-plot pubescence test, 56 gray pixels, 41 light tawny pixels, and 76 tawny pixels (Figures 4(b) and 4(c)). The final output of the model was tested as both pixel counts (Figure 4(d)) and a majority pixel classification per plot (Figure 4(e)). The gray pubescence could be clearly distinguished from the light tawny and tawny pixels in a range of ML methods, including the chosen SVM model. Light tawny and tawny were difficult to distinguish in the final method, where light tawny plots contained an even number of light tawny and tawny pixels, while tawny plots had a majority of pixels classified as tawny by the SVM model. No gray plots were misclassified, and gray pixels were uncommon in tawny and light tawny plots.

**Figure 5:**
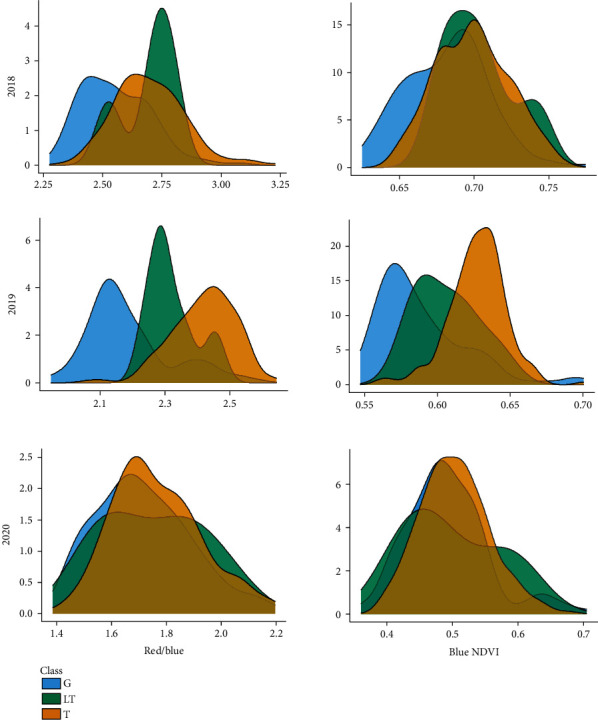
Distributions of three years (2018, 2019, and 2020) of mature soybean plots (Full Field images) imaged prior to harvest shown with two plot-mean indices (red/blue and blue NDVI), where each plot had genetic data available to estimate pubescence types (gray, light tawny, or tawny).

The plot genotype data was joined to the image data to determine the expressed pubescence for each plot in the three-season images, finding that the 2018 and 2020 indices did not correspond well with the estimated pubescence type (Figure 5). The 2019 data (from the same field as the 60-plot pubescence test) showed class distinction, indicating that the images were not sufficient in 2018 and 2020 to properly distinguish the classes. The red/blue ratio showed the most distinct separation of classes, though in all cases, there was overlap in the tails of each of the distributions. For the ML generalization to the 2018, 2019, and 2020 field images, a similar trend was observed, whereas the 2019 image reflected the trained model well, and the 2018 and 2020 images were not accurately classified by the SVM (Figure S1).

## 4. Discussion

A comparison of the hyperspectral to the aerial image data found that similar trends are present in both datasets. From the initial analysis of both the hyperspectral and aerial data, it was determined that the classification of soybean pubescence would be possible from the imagery in a high-throughput method. The ability to quickly discriminate the pubescence types is a valuable tool for soybean researchers, even if the light tawny class is difficult to distinguish from the other two classes. Based on the pixel-based spectral classification, the discrimination between tawny and light tawny is unclear even in ideal circumstances; however, the gray class is clearly distinguishable from the light tawny and tawny classes. This can provide a rapid screen for breeding purposes, allowing for resource-intensive manual phenotyping to be carried out in later-stage field trials when knowledge of exact pubescence phenotype is desired or required.

The models and indices were established based on data from the 2019 growing season and generally performed well on plots from the same image that were not in the training data. It was determined that they did not generalize well to the 2018 and 2020 late-season imagery. There are several possibilities that could explain the poor model inference, including spatial resolution, sensor differences, imaging/flight conditions, and single-year training data from a small number of genotypes, which may not represent the entire range of variation for the pubescence classes. The lack of robustness for these analyses over multiple field seasons while using calibrated imagery may limit the application of these methods to imagery without an internal reference panel.

Spatial resolution was a concern for capturing enough pixels for each plant. The images that worked for both the indices and ML had smaller pixels, meaning that when plant pixel data was extracted from the full image, the extracted pixels represented only plant material. This leads to the hypothesis that low spatial resolution could make distinguishing the classes difficult. In the case of the lower resolution imagery (2018 and 2020), there is a greater chance that the plant pixels were mixed with soil, as the spatial resolution was not high enough to clearly distinguish soil and plant pixels [30, 31]. This means that the plant color information does not exist alone within the remaining plant pixels to sufficiently classify the pubescence type. A recommendation for capturing imagery to classify soybean pubescence would be to ensure sufficient spatial resolution to obtain pure plant pixels, for example, flying at a lower altitude than typically used to capture in-season canopy data to increase spatial resolution.

Due to field limitations, the pubescence test used for the training data was only grown in the 2019 growing season. The genotypes were chosen because of the high confidence in their pubescence type from multiple years of trials and in several cases, variety registration. Given that the focus was on cultivars and experimental genotypes that had such a high level of scrutiny for their pubescence type, the test size was limited to 60 entries for which the pubescence data was available and reliable. The size of the pubescence test could be a limitation for representing the true variation for each of the pubescence types. With additional genetic data, it might be possible to use larger field tests for training data, but concerns with plot purity increase in large tests for generating model training data.

A possibility for improved pubescence classification accuracy and model generalization is the exploration of spatially aware ML models [32]. Several options exist which could be tested, including spatially aware pixel classifiers, which would use information from neighbouring pixels to help improve classification accuracy [33]. Another option would be to use convolutional neural nets (CNNs) where entire plot images are used for classification, which was recently used for estimating soybean maturity [6]. However, the training data requirements for such a model are much larger than the SVM classifier used here. An additional benefit for a CNN approach would be that soil context would be captured whereas it might be confounding the pubescence color when images are of lower spatial resolution, and a CNN could also capture variation in linked canopy traits and spatial distribution of plant pubescence pixels which are not captured by pixel classification methods. However, making a CNN generalize well would require diverse training data from multiple years and locations and a wide range of germplasm to properly capture the total variation in the pubescence classes.

An SVM model appears to be appropriate for inference within an image mosaic from one UAS flight, meaning that the outlined methodology used here could be applied by any breeding program provided that plots of training data of known pubescence types are grown in each field. This is not an onerous requirement given the need for border and filler plots within a large research field. A model can be trained for each image, and pubescence types can be estimated for the entire field. This workflow is much simpler, faster, and completely objective compared to visual assessment. Even if light tawny types are difficult to distinguish from tawny types, being able to confidently distinguish gray from tawny and light tawny is similar in accuracy to current visual assessment methods, at a much lower cost and higher throughput while maintaining the model objectively.

An important finding of this work is that a simple red/blue ratio works well for gray vs. tawny and light tawny classification. This could eliminate the need for imagery with red edge and near-infrared spectral bands to classify pubescence, which reduces the cost barrier for research programs without access to 5-band imagery. Again, with the similar previously described caveats of sufficient spectral resolution, a simple ratio is easy for non-GIS experts to calculate and generate pubescence data from large field images.

Ongoing work is required to identify additional effects on the application of these indices and ML models such as background, maturity, year, and sensor effects. This work was conducted on early-maturing soybean genotypes, and there may be additional interactions in later-season soybeans that are not captured by the images used here. Ideally, a long-term trial from various maturities and soil types and with a range of genetic backgrounds could be used to generate a more robust model to explain soybean pubescence from imaging data.

In conclusion, several indices and an SVM model were tested to characterize soybean pubescence. Both indices were able to distinguish the gray and tawny pubescence types, and the SVM model was able to differentiate gray compared to combined light tawny and tawny. The indices and models did not generalize across field seasons and imaging platforms even using calibrated imagery which may limit the application of this technique. Further work could expand on the presented data with additional years, locations, and maturities to further characterize the variation in pubescence.

## Figures and Tables

**Table 1:** Sensor and imaging equipment.

	2018 and 2019	2020
Drone	DJI M-210	DJI P4 Multispectral RTK
Camera	MicaSense RedEdge3 (2018) MicaSense Altum (2019)	Built-in
Additional equipment	Timble APX-15-E1 (2019)	N/A
Blue	475 nm ± 16 nm	450 nm ± 16 nm
Green	560 nm ± 13.5 nm	560 nm ± 16 nm
Red	668 nm ± 7 nm	650 nm ± 16 nm
Red edge	717 nm ± 6 nm	730 nm ± 16 nm
Near-infrared	842 nm ± 28 nm	840 nm ± 26 nm
Ground sample distance	3.9 cm (2018), 2.6 cm (2019)	3.6 cm (2020)

**Table 2:** Jeffries-Matusita distance for five-band (B, G, R, RE, and NIR) and subset three-band (B, G, and R) images to determine the spectral separability between the pubescence classes in the 60-plot training image. Higher values suggest better class separability.

Class pair separation	5 bands	3 bands	Difference
Tawny:light tawny	1.90333	1.86054	-0.04279
Tawny:gray	1.90956	1.86714	-0.04242
Light tawny:gray	0.78328	0.33899	-0.44429

## Data Availability

The imagery data used to support the findings of this study are available from the authors upon request.

## Supplementary Materials

Supplementary Materials Supplementary data on the field imagery, PCA loadings, SVM classification, and numerical index data.
