# Supplementary material for: Classification of Soybean Pubescence from Multispectral Aerial Imagery
Source: Plant Phenomics. 2021 Aug 4;2021:9806201. doi: 10.34133/2021/9806201 (PMC8363756; doi:10.34133/2021/9806201)
Supplement: Supplementary Materials — Supplementary data on the field imagery, PCA loadings, SVM classification, and numerical index data. [file 9806201.f1.docx]

**Supplementary Material**

**Description**: Supplementary data on the field imagery, PCA loadings, SVM classification and numerical index data.

**Table S1**. Field images used for studying soybean pubescence in 2018, 2019 and 2020 at the Elora Research Station, University of Guelph. Plots were 1.6m x 5m, with the number of plots indicated the number of plots from which data was collected for that year, which was lower than the total number of plots in the test due to some genotypes having poor germination and poor stand quality. Full Field pubescence was inferred via SNP markers associated with the known pubescence genes. Reference Test pubescence was determined from long-term breeding program records and previous multi-year testing for late-stage

| **Growing Season** | **Image** | **Total Plots** | **Tawny Plots** | **Light Tawny Plots** | **Gray Plots** | **Notes** |
| --- | --- | --- | --- | --- | --- | --- |
| 2018 | Full Field 2018 | 366 | 224 | 12 | 129 | Originally 400-plot test (2 replicate x 200 genotypes), 34 plots were dropped due to poor emergence. |
| 2019 | Full Field 2019 | 344 | 216 | 6 | 122 | From seed quality issues identified in 2018, plot numbers were reduced in 2019 |
| 2019 | Reference Test 2019 | 60 | 21 | 15 | 24 | Reference set of soybean genotypes with long term field verification of pubescence. Set up as a 3 replicate x 20 genotype test. |
| 2020 | Full Field 2020 | 344 | 216 | 6 | 122 | Same genotypes as 2019 |

**Table S2**. Loadings from the principal components analysis of 36,039 pixels used in Figure 2C. Band 1 through Band 5 represent the green, blue, red, red-edge and near-infrared bands from the aerial imagery, respectively.

|  | PC1 | PC2 | PC3 | PC4 | PC5 |
| --- | --- | --- | --- | --- | --- |
| Band 1 | 0.429 | 0.694 | -0.335 | 0.281 | -0.379 |
| Band 2 | 0.455 | 0.300 | 0.023 | -0.516 | 0.660 |
| Band 3 | 0.458 | -0.136 | 0.588 | 0.618 | 0.208 |
| Band 4 | 0.457 | -0.249 | 0.330 | -0.503 | -0.606 |
| Band 5 | 0.437 | -0.590 | -0.657 | 0.139 | 0.099 |


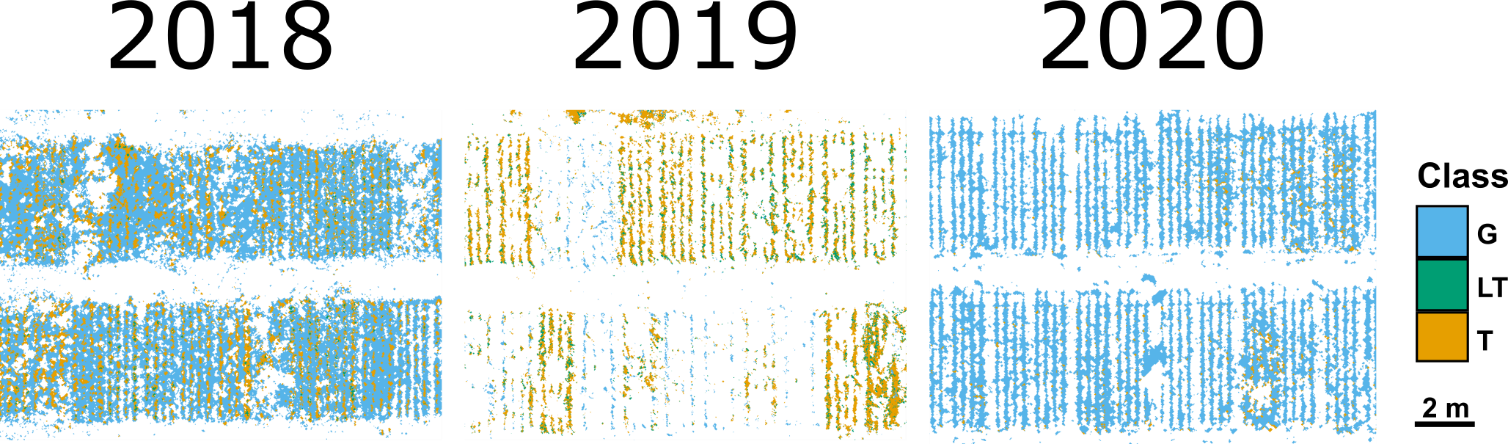


**Figure S1.** Support vector machine classification output from end-of-season images from 2018, 2019 and 2020. 2018 and 2020 did not have effective classification from the SVM model, and the model performed well on the 2019 data from the same image on which it was trained.

**Table S3.** (a) Means, lower (2.5%) and upper (97.5%) confidence intervals (CI) for the Blue NDVI and Red/Blue indices from the 60-plot pubescence test, calculated from the ANOVAs for each index. (b) Calculated breakpoints using the midpoint between CIs for class separation to generalize the indices.

A)

| Index | Class | Lower CI | Mean | Upper CI |
| --- | --- | --- | --- | --- |
| Blue NDVI | G | 0.570 | 0.576 | 0.582 |
|  | LT | 0.642 | 0.658 | 0.673 |
|  | T | 0.667 | 0.682 | 0.696 |
| Red/Blue | G | 2.376 | 2.426 | 2.476 |
|  | LT | 2.816 | 2.947 | 3.079 |
|  | T | 3.027 | 3.151 | 3.275 |

B)

| Index | G | LT | T |
| --- | --- | --- | --- |
| Blue NDVI | < 0.612 | 0.612 to 0.670 | > 0.670 |
| Red/Blue | < 2.646 | 2.646 to 3.053 | > 3.053 |
